# Genetic and Genomic Analysis of Systemic Sclerosis: A Narrative Review of the Recent Advancement

**DOI:** 10.31662/jmaj.2025-0167

**Published:** 2025-11-14

**Authors:** Yuki Ishikawa, Chikashi Terao

**Affiliations:** 1 Laboratory for Statistical and Translational Genetics, Center for Integrative Medical Sciences, Yokohama, Japan; 2 Clinical Research Center, Shizuoka General Hospital, Shizuoka, Japan; 3 Department of Applied Genetics, The School of Pharmaceutical Sciences, University of Shizuoka, Shizuoka, Japan

**Keywords:** systemic sclerosis, genome-wide association study (GWAS), transcriptome, single-cell RNA sequence (scRNA-seq), integrative multi-omic study

## Abstract

Systemic sclerosis (SSc) is one of the systemic autoimmune diseases characterized by disease-specific autoantibodies and generalized fibrosis in connective tissues and internal organs, resulting from microvascular and immune dysfunctions, which lead to premature death in affected individuals. The etiology of SSc is complex and poorly understood; however, as with most autoimmune conditions, it is widely accepted that both environmental and genetic factors interact and contribute to disease development.

Over the last decade, genome-wide association studies (GWAS) have identified multiple genetic markers associated with SSc, and a number of causal variants have also been fine-mapped using state-of-the-art statistical techniques. Furthermore, the latest East Asian GWAS identified novel risk variants that were not as strongly represented in Europeans as in East Asians, and also provided additional novel risk variants, highlighting the importance of enrolling a diverse population for the analysis of complex traits. The association of human leukocyte antigen regions has been clarified mainly in individuals of European descent, with the identification of clinical subtype-specific associations of certain alleles. The associations in East Asian SSc are currently being analyzed by our team.

Recent advances in single-cell ribonucleic acid (RNA) sequence technology have enabled the identification of transcriptomic changes at the single-cell level in a cell type or tissue-specific manner, allowing for the identification of disease-relevant cells or transcriptomes. To date, multiple single-cell RNA sequence studies have been conducted, primarily focusing on cells and transcriptomes in peripheral blood, skin, or lung.

Despite the remarkable advances in state-of-the-art technology for both genetics and transcriptomics, gene expression regulations, especially by enhancers, enriched for disease heritability and thus critical for SSc development, remain unresolved. Integrative analysis using multi-omics approaches, combined with deep phenotyping of study cohorts, is imperative to fully characterize the genetic component of this disease and to identify causal variants, which may lead to more targeted and effective treatment of SSc.

## Introduction

Systemic sclerosis (SSc) is a systemic autoimmune disease characterized by generalized fibrosis in connective tissues and internal organs as a consequence of microvascular and immune dysfunctions, which lead to premature death in affected individuals. Females are predominantly affected, with onset typically between the ages of 30 and 50, although SSc can occur at any age ^(1)^.

Clinically, SSc is divided into limited cutaneous SSc (lcSSc) and diffuse cutaneous SSc (dcSSc) ^(2)^. SSc is also classified based on serum autoantibody profiles, particularly anti-centromere antibodies (ACAs), and anti-topoisomerase antibodies (ATAs), which are found in approximately 50%-70% of patients ^(3)^. In most cases, ACA and ATA are mutually exclusive, and each autoantibody is associated with a distinct clinical phenotype; ACA is typically associated with lcSSc, uncommon pulmonary fibrosis (PF), late-onset pulmonary hypertension, and generally a favorable prognosis; whereas ATA is a marker for dcSSc and clinically significant PF, resulting in a poorer prognosis than the ACA(+) subtype ^(4)^. In addition to these classical classifications, a newer classification based on pathological processes has emerged, categorizing SSc into a vascular phenotype and an immune-fibrotic phenotype. The vascular phenotype is characterized by one or more features, including Raynaud’s phenomenon, digital ulcers, pulmonary arterial hypertension, and scleroderma renal crisis, and is caused by vascular damage and dysfunction ^(5)^. The fibrotic phenotype, on the other hand, results from the excessive production of collagen and extracellular matrix proteins, leading to tissue and organ fibrosis due to a complex interplay between a dysregulated immune system and vascular injury. Essentially, vascular damage precedes and contributes to the development of fibrosis in SSc.

It is widely accepted that SSc is a complex and multifactorial disease in which genetic risks and environmental factors interact to trigger disease onset ^(6)^, as seen in other autoimmune diseases such as rheumatoid arthritis (RA). Despite recent progress in disease management ^(7)^, SSc still presents high morbidity and mortality mainly due to a poor understanding of its underlying pathophysiological mechanisms ^(1)^.

### Heritability of SSc

Feghali-Bostwick et al. ^(8)^ performed a classical twin study and found similar SSc concordance rates between monozygotic and dizygotic twins, suggesting low heritability of SSc. However, this study included a very limited number of twins, and the subjects had a wide range of ages. In contrast, Arnett et al. ^(9)^ showed that first-degree relatives of SSc patients had a 13-fold higher risk of developing SSc compared to the general population, and a 15-fold higher risk among siblings of SSc patients. Moreover, they reported an increased familial risk of developing other autoimmune diseases such as hypothyroidism, hyperthyroidism, systemic lupus erythematosus (SLE), or certain SSc manifestations, including Raynaud’s phenomenon or interstitial lung disease ^(9)^. These studies indicate a significant contribution of genetic components to SSc development and suggest shared genetic components between SSc and other autoimmune diseases.

Bossini-Castillo et al. ^(10)^ estimated SSc heritability using a mixed model (genomic-relatedness-based restricted maximum-likelihood, GREML) ^(11)^ applied to two large-scale genome-wide association studies (GWAS) ^(12), (13)^. While they found that the heritability (liability-scale) of SSc is lower than that of other autoimmune diseases, such as inflammatory bowel disease (IBD) or type 1 diabetes ^(11), (14)^, they also confirmed that the twin concordance study mentioned above ^(8)^ had underestimated heritability of SSc ^(10)^.

Although it has been indicated that the heritability of SSc might be lower than that of other autoimmune diseases ^(10)^, it is evident that the genetic component plays an important role in the pathogenesis of SSc. Thus, estimating precise heritability and clarifying the genetic roles in pathogenesis are of future interest in the genetic study of SSc.

## Materials and Methods

The most frequent type of DNA variation is a single-nucleotide polymorphism (SNP). Two basic approaches often used for genetic association studies are the candidate gene approach and GWAS. Both modalities identify SNPs and determine the likelihood that a variant occurs more or less frequently in cases than in controls. For both approaches, the association is first tested in a discovery cohort and then replicated in a non-overlapping group of cases and controls. GWAS scans the entire genome for SNPs associated with a certain disease without any assumptions and is therefore considered an unbiased approach. Indeed, GWAS has enabled the identification of new loci that would not have been discovered otherwise ^(15)^. However, because most GWAS provide approximately 80% coverage for common polymorphisms in the human genome, they often miss unusual or rare variants ^(6)^. Moreover, GWAS usually investigates SNPs that are in strong linkage disequilibrium with other SNPs and serve as proxies for them; thus, the SNPs identified by GWAS are regarded as tags for the yet-to-be-identified causal alleles. The immunochip array is another type of SNP genotyping tool with high-density mapping of numerous variants, including those known to be associated with target diseases or phenotypes ^(13), (16)^. It also includes several rare variants believed to have significant functional effects that might have been previously overlooked ^(17)^. This approach has recently shown promising results in various autoimmune and immune-related diseases, including SSc ^(13), (16)^. These studies have resulted not only in the identification of susceptibility genes but also in the refinement of the associations in previously reported risk loci for large cohorts at reduced costs. Moreover, these reports increased the number of shared genetic markers among different disorders, further supporting the common genetic component of autoimmunity.

### Preceding GWAS for SSc

A total of six large-scale GWASs have been conducted (Table 1), and 33 risk loci have been identified thus far (Table 2).

**Table 1.** Large-scale GWASs for SSc.

Author	Study	Study model	Target phenotype(s)	Population	Case	Ctrl	Independent signals	Novel loci	Reference
Radstake, et al	GWAS	Logistic regression model	SSc	EUR	5049	10220	2	2	(12)
Gorlova et al	GWAS	Logistic regression model	lcSSc, dcSSc, ACA, ATA	EUR	5471	10142	1 (lcSSc)	1 (lcSSc)	(4)
Allanore et al	GWAS	Logistic regression model	SSc	EUR	2246	5702	0	0	(18)
Terao et al	GWAS, GWAS meta-analysis	Logistic regression, inverse variance fixed effect model	SSc	EAS, EUR	EAS 1280, EUR 3156	EAS 3660, EUR 11091	EAS-EUR 3	EAS-EUR 2	(19)
López-Isac et al	GWAS meta-analysis	inverse variance fixed effect model	SSc	EUR	9095	17584	28	13	(20)
Ishikawa et al	GWAS, GWAS meta-analysis	Logistic regression, inverse variance fixed effect model	SSc, lcSSc, dcSSc, ACA, ATA	EAS, EUR (Summary statistics)	1428	112599	EAS 6; EAS-EUR 30	EAS 3; EUR-EAS 3	(21)
Pu et al	Exome-wide association study	Logistic regression model	SSc	EAS	1006	6120	6	2	(22)

Studies with both cases and controls >1000 are listed; signals and loci are outside the MHC region. ACA: anti-centromere antibodies; ATA: anti-topoisomerase antibodies; dcSSc; diffuse cutaneous systemic sclerosis; EAS: East Asia; EUR: Europeans; GWAS: genome-wide association study; lcSSc: limited cutaneous systemic sclerosis; MHC: major histocompatibility complex; SSc: systemic sclerosis.

**Table 2.** Known Genome-Wide Significant SSc Risk Loci Outside the MHC Region.

ID	CHR	BP (GRCh37)	Location	Gene locus	EA	NEA	OR	95% CI		P	Population
rs3790566	1	67814440	Intronic	*IL12RB2*	T	C	0.86	1.11	1.22	3.8E-10	EUR
rs6697139	1	161660696	Intergenic	*RPL31P11-FCRLA*	T	G	2.05	1.66	2.54	4.9E-11	EAS
rs2056626	1	167420425	Intronic	*CD247*	G	T	0.81	0.76	0.86	1.3E-11	EUR
rs1857066	1	173332629	ncRNA intronic	*LOC100506023*	A	C	0.87	0.83	0.91	5.0E-09	EUR
rs16832798	2	191534372	Intronic	*NAB1*	C	T	0.85	0.80	0.90	5.2E-09	EUR
rs3821236	2	191902758	Intronic	*STAT4*	A	G	1.31	1.24	1.39	1.9E-23	EUR, EAS
rs398390	3	28074673	Intergenic	*LINC01980-CMC1-EOMES*	C	T	1.12	1.07	1.16	1.7E-08	EUR-EAS
rs4076852	3	58375286	Intronic	*FLNB-DNASE1L3-PXK*	G	A	1.16	1.11	1.22	1.0E-10	EUR
rs9884090	3	119116150	Intronic	*POGLUT1-TIMMDC1-CD80-ARHGAP31*	A	G	0.83	0.79	0.88	1.9E-10	EUR
rs589446	3	159733527	ncRNA intronic	*IL12A-AS1*	T	G	0.86	0.82	0.90	2.0E-10	EUR
rs11724804	4	965779	Intronic	*DGKQ*	A	G	1.17	1.11	1.22	5.3E-11	EUR
rs230534	4	103449041	Intronic	*NFKB1*	T	C	1.15	1.10	1.21	5.4E-09	EUR
rs4317244	4	186320906	Exonic	*LPR2BP*	G	C	1.53	1.31	1.79	4.2E-08	EAS
rs3792783	5	150455732	Intronic	*TNIP1*	G	A	1.20	1.14	1.27	2.4E-12	EUR
rs4134466	6	106577368	Intergenic	*PRDM1*	G	A	1.17	0.81	0.90	6.6E-10	EAS-EUR
rs633724	6	106734040	Intronic	*ATG5*	T	C	1.13	1.09	1.18	2.8E-09	EUR
rs5029949	6	138197506	Intronic	*TNFAIP3*	G	A	1.50	1.32	1.69	1.7E-10	EAS
rs10484921	6	152042260	Intronic	*ESR1*	A	C	1.15	1.09	1.20	8.3E-09	EUR-EAS
rs36073657	7	128651522	Intronic	*IRF5-TNPO3*	T	C	1.40	1.31	1.51	3.1E-21	EUR
rs2736340	8	11343973	Intergenic	*FAM167A-BLK*	C	T	0.81	0.77	0.84	3.3E-21	EUR
rs685985	8	61564964	Intergenic	*RAB2A-CHD7*	T	A	0.87	0.82	0.91	3.8E-08	EUR
rs6598008	11	618172	Intronic	*CDHR5-IRF7*	A	G	0.80	0.74	0.87	2.0E-08	EUR
rs2651804	11	2348619	Intergenic	*TSPAN32-CD81-AS1*	T	C	0.82	0.77	0.87	2.5E-10	EUR
rs11217020	11	118639353	Intronic	*DDX6*	A	G	0.84	0.80	0.89	2.1E-11	EUR
rs2819422	14	105408955	Exonic	*AHNAK2*	G	A	0.76	0.70	0.82	2.7E-11	EAS
rs45471499	14	106321330	Exonic	*IGHM*	A	G	1.55	1.35	1.79	1.2E-09	EAS
rs1378942	15	75077367	Intronic	*CSK*	C	A	1.18	1.13	1.23	1.8E-14	EUR
rs11117420	16	85971922	Intronic	*IRF8*	C	G	0.81	0.76	0.85	3.8E-15	EUR
rs883770	17	38063381	Intronic	*IKZF3-GSDMB*	T	C	1.13	1.08	1.17	4.8E-09	EUR
rs3894194	17	38121993	Misssense	*GSDMA*	A	G	1.18	0.80	0.89	1.4E-10	EAS-EUR
rs1005714	17	73224639	Intronic	*NUP85-GRB2*	G	C	0.85	0.81	0.90	1.9E-08	EUR
rs2305743	19	18193191	Intronic	*IL12RB1*	A	G	0.83	0.78	0.88	4.6E-10	EUR
rs9074	20	44688665	UTR3	*SLC12A5*	A	G	1.12	1.08	1.17	2.2E-08	EUR-EAS

AHNAK2: AHNAK nucleoprotein 2; ATG5: autophagy related 5; CD247: CD247 molecule; CHR: XXX; CSK: C-terminal src kinase; DDX6: DEAD-box helicase 6; DGKQ: diacylglycerol kinase theta; EA: effect allele; EAS: East Asia; ESR1: estrogen receptor 1; EUR: Europeans; FAM167A-BLK: family with sequence similarity 167 member A - B lymphoid tyrosine kinase; FLNB-DNASE1L3-PXK: filamin B - DNASE1-like 3 - PX domain containing serine/threonine kinase; GSDMA: gasdermin A; IGHM: immunoglobulin heavy constant mu; IKZF3-GSDMB: IKAROS family zinc finger 3 - gasdermin B; IL12A-AS1: IL12A antisense RNA 1; IL12RB1: interleukin 12 receptor subunit beta 1; IL12RB2: interleukin 12 receptor subunit beta 2; IRF5-TNPO3: interferon regulatory factor 5 - transportin 3; IRF8: interferon regulatory factor 8; LINC01980-CMC1-EOMES: long intergenic non-protein coding RNA 1980 - CMC1 - eomesodermin; LOC100506023: uncharacterized LOC100506023; LRP2BP: LDL receptor-related protein 2 binding protein; MHC: major histocompatibility complex; NAB1: NGFI-A binding protein 1; NEA: non-effect allele; NFKB1: nuclear factor kappa B subunit 1; NUP85-GRB2: nucleoporin 85 - growth factor receptor bound protein 2; POGLUT1-TIMMDC1-CD80-ARHGAP31: protein O-glucosyltransferase 1 - TIMMDC1 - CD80 - Rho GTPase activating protein 31; PRDM1: PR/SET domain 1; RAB2A-CHD7: RAB2A member RAS oncogene family - chromodomain helicase DNA binding protein 7; CDHR5-IRF7: Cadherin related family member 5 - interferon regulatory factor 7; RPL31P11-FCRLA: ribosomal protein L31 pseudogene 11 - Fc receptor like A; SLC12A5, solute carrier family 12 member 5; SSc: systemic sclerosis; STAT4: signal transducer and activator of transcription 4; TNFAIP3: tumor necrosis factor alpha-induced protein 3; TNIP1: TNFAIP3 interacting protein 1; TSPAN32-CD81-AS1: tetraspanin 32 - CD81 antisense RNA 1.

The pioneering GWAS for SSc was conducted in individuals of European descent. Five GWASs and one East Asian exome-wide sequence study followed thereafter ^(4), (18), (19), (20), (21), (22)^. The study employed a two-step approach: a discovery dataset of 2,296 SSc patients and 5,171 controls, and a replication dataset of 2,753 SSc and 5,049 controls. Three SNPs were identified as genome-wide significant (p > 5 ´ 10^-8^) index SNPs, namely rs3821236 in an intron of *STAT4*, rs10488631between *TNPO3-IRF5*, rs6457617 between *HLA-DQB1* and *HLA-DQB2*, and rs2056626 in an intron of* CD247* (Table 1). All loci identified in this study were replicated in subsequent studies, despite sample overlaps, suggesting a robust association of these loci in SSc subjects of European descent.

Association analyses of the main clinical subtypes of SSc, lcSSc, dcSSc, ACA, and ATA were then conducted in the same samples and reported separately by Gorlova et al. ^(4)^. In this study, GWAS was conducted by comparing SSc subjects with one of the main clinical phenotypes to non-SSc control samples. A genome-wide significant association was identified between lcSSc and rs11642873 near IRF8, and suggestive associations (p £ 1.0 ´ 10^-5^) were identified for lcSSc with rs12540874(intronic, *GRB10*), for dcSSc with rs11171747 (upstream, *RPL4/ESYT1*), and for ACA with rs11047102 (intronic, *SOX5*). In the major histocompatibility complex (MHC) region, genome-wide significant associations were identified between *HLA-DQB1* and ACA, *HLA-DPA1/B1* and ATA, and *NOTCH4* with ACA or ATA. The study showed that the distinctive clinical phenotypes can be explained by different genetic backgrounds.

Allanore et al. ^(18)^ conducted a two-step study using a French discovery dataset consisting of 564 SSc patients and 1,776 non-SSc controls, and a European replication dataset consisting of 1,682 SSc patients and 3,926 non-SSc controls. The French discovery dataset identified several candidate risk SNPs, which were examined in the replication dataset, leading to the identification of suggestive associations of *HLA-DQB1* and *PSORS1C1* in the MHC region, and *TNIP1* and *RHOB* in the non-MHC region*.* Intriguingly, the French dataset did not show significant associations with the known risk loci in the *STAT4*, *CD247*, and *TNPO3*-*IRF5* regions, while the non-French replication dataset showed significant associations in these regions, suggesting a potential difference in genetic architecture between French and non-French populations.

We conducted the first East Asian GWAS for SSc by enrolling 1,280 SSc patients and 3,360 non-SSc controls from multiple centers in Japan ^(19)^. *STAT4(*rs12612769, p = 4.7 ´ 10^−8^*)* and *HLA-DRA* (rs9268636, p = 9.6 ´ 10^−10^) were replicated for their significant association in Japanese SSc. The meta-analysis with the French dataset identified an enhanced association of the *STAT4* locus （2_191955205, p = 3.0 ´ 10^−11^, further confirming the global association of the *STAT4* locus in SSc. On the other hand, the associations in the MHC regions were attenuated in the meta-analysis, indicating a distinctive pattern of MHC association between Japanese and French SSc populations. In addition, a replication study was conducted for 33 candidate loci identified in the meta-analysis, using independent datasets from Japanese (564 SSc and 1,863 non-SSc controls) and European populations (1,582 SSc and 6,694 non-SSc controls; 101 SSc and 2,621 non-SSc controls), This led to the identification of novel susceptibility loci in *GSDMA* (rs3894194, p = 1.4 ´ 10^−10^) and *PRDM1* (rs4134466, p = 6.6 ´ 10^−11^). Multiple genes, including *LRRC3C*, *ORMDL3*, and *GSDMB*, are mapped to the genomic region near *GSDMA*, and several SNPs in this region have been identified as susceptibility signals for RA and IBD. However, rs3894194 is not in high linkage disequilibrium (LD) with those SNPs (r2 < 0.38), indicating an independent association of rs3894194. *PRDM1* encoding BLIMP1 is known for its association with RA, SLE, and IBD and is located close to *ATG5*. Intriguingly, rs9373839 in ATG5 has been identified as a risk SNP in European SSc, while rs9373839 and rs3894194 are not in high LD (r2 < 0.15). These SNPs were found to be independently associated through conditional analysis, highlighting that nearby genes, *PRDM1* and *ATG5*, are independently associated with SSc.

### The largest European GWAS for SSc

The largest-ever European GWAS was reported in 2019 ^(20)^. The study comprised 9,095 SSc patients and 17,584 non-SSc control of European descent, obtained by expanding the previous European GWAS. A total of 27 independent signals, including 13 novel susceptibility signals, were identified in the non-MHC region. A subsequent Bayesian fine-mapping analysis using PAINTOR successfully narrowed down the number of causal SNPs (<5) in 12 loci. Most of the fine-mapped variants with a posterior probability of inclusion (>0.1) were located in promoter or enhancer regions, and 75.83% (61/81) of them were expression quantitative trait loci (eQTL).

By referring to publicly available experimental data, such as chromatin immunoprecipitation (ChIP)-sequence (H3K27ac), chromatin interaction analysis by ChIP with Hi-C (HiChIP) or promoter capture Hi-C, the study also identified SNPs in the promoter regions of *CD247*, *IL12RB2*, *NFKB1*, *STAT4*, *IRF8* were overlapped with promoter-enhancer interaction in CD4+T cells, B cells, or macrophages.

Taking advantage of the large sample size, the study also investigated potential associations specific to the major clinical subtypes of SSc, lcSSc, dcSSc, ACA, and ATA, through case-control studies. They identified 18 and 5 genome-wide significant signals for lcSSc and dcSSc, respectively. Among these associations, those between lcSSc and *MERTK*, and dcSSc and *ANKRD12,* were subtype-specific. *MERTK* encodes a MER/AXL/TYRO3 receptor tyrosine kinase and is known for its association with multiple sclerosis ^(23)^. rs4798783 in *ANKRD12* is an eQTL in fibroblasts, and TWSG1 is known to enhance transforming growth factor β (TGFβ) signaling. ACA was associated with nine signals, while only one was significantly associated with ATA. Due to the limited sample size, no significant association signal was identified for the subtype with RNA polymerase III. Notably, although many signals were novel for the corresponding clinical subtypes, most were also significantly associated with global SSc. Hence, subtype-specific signals were limited to those in *MERTK* and *ANKRD12*.

### The largest East Asian GWAS for SSc

We recently conducted the largest East Asian GWAS, comprising 1,428 cases and 112,599 controls ^(21)^. The study identified five genome-wide significant loci, including three novel association signals that had not been reported previously in East Asian SSc subjects. We also conducted a cross-ancestry meta-analysis with the latest European meta-GWAS and identified 30 genome-wide significant loci, three of which had never been reported in association with SSc. We then fine-mapped the SNPs in the genome-wide significant loci for both Japanese GWAS and the cross-ancestry meta-analysis, and successfully narrowed down the number of candidate causal variants in the 95% credible sets for several loci. Among such loci, one of the novel significant loci identified in the Japanese GWAS, namely the FCGR-FCRL gene cluster region, contained four candidate causal variants, including the lead SNP, rs6697139, in its 95% credible set. The lead SNP, rs6697139, and its complete LD SNP, rs10917688, had almost identical posterior inclusion probabilities, and the latter was found to be located in the enhancer region, which was further revealed to be a part of the binding motif of IRF8. The enhancer-related histone marks, H3K27ac and H3K4me1, were identified in primary CD8^+^ memory T cells and primary B cells, respectively, at this locus, implying a cell-type-specific effect of the SNP. As the IRF8 locus was one of the genome-wide significant risk loci in European SSc, we investigated a potential interactive effect between rs10917688 and the lead SNP in IRF8, rs11117420. Intriguingly, the association of rs10917688 was significant only in the presence of the risk allele of rs11117420, suggesting an epistatic association between rs10917688 and rs11117420. We further identified significant heritability enrichment in active histone marks in B cells in both Japanese and European SSc, underscoring the roles of B cells in SSc development. The SNP-based heritability estimates were not as high as those of other autoimmune diseases such as RA and SLE; however, we confirmed that a substantial proportion of heritability is explained by SNPs, thereby supporting the polygenic nature of the disease. Based on the observed polygenicity, we constructed a polygenic risk score (PRS) and tested its disease-predictive performance. As expected, the predictive performance, represented by the area under the curve of the PRS model (logistic regression), was not high enough for clinical implementation. However, we observed improvement in performance by prioritizing the lead SNPs from the cross-ancestry meta-analysis and the top 5% SNPs for IRF8 biding in blood B cells, as annotated by IMPACT, further supporting the shared polygenic nature of SSc and underscoring the pathogenic roles of B cells in SSc.

### Association of human leukocyte antigen genes

As seen in other autoimmune diseases, strong associations of human leukocyte antigen (HLA) genes with SSc susceptibility have been reported mainly in individuals of European descent.

The MHC region is one that spans about 7.6 sMb on chromosome 6 and is densely packed with many genes. These genes encode not only HLA molecules but also non-HLA, which are functionally distinct from HLAs. On the other hand, some non-HLA genes show HLA gene dependency, as seen in the epistatic relationship between HLA-B51 and ERAP1. Furthermore, the MHC region has a more complex LD structure compared to non-MHC regions. Hence, association studies of the MHC region need to be conducted separately from those of non-MHC regions, using different strategies for fine-mapping the region to identify plausible causal variants.

Previous large-scale European GWASs identified that SNPs in the* HLA-DQB1* genes (rs6457617, rs9275224, rs6457617, rs9275245) are strongly associated with SSc susceptibility ^(4), (12), (13), (18), (24)^. Among the clinical subtypes, ACA showed stronger associations with rs645761 and rs9275390, while ATA presented stronger associations with rs3129882 in *HLA-DRA*, rs3129763 in *HLA-DQA1/DRB1*, and rs987870 in *HLA-DPA1/DPB1* ^(4)^. In addition to SNPs, associations of HLA-DPB1*13:01 with ATA, HLA-DQB1*05:01, and *HLA-DQB1* alleles encoding a non-leucine residue at position 26 (DQB1*26 epi) with ACA, and HLA-DRB1*04:04, HLA-DRB1*11, and HLA-DQB1*03 with ARA were reported by Arnett et al. ^(25)^. Furthermore, they also reported that African American showed distinctive allele associations DRB1*08:04, and DQA1*05:01, DQB1*03:01, indicating population-specific associations ^(19)^.

The largest association analysis of the HLA region was conducted on the European GWAS samples described above ^(26)^. The study confirmed the associations of HLA-DRB1*11:04 and HLA-DPB1*13:01 and also identified a novel association with HLA-B*08:01. Regarding clinical subtypes, the study identified specific associations of HLA-DQA1*02:01 and HLA-DQA1*05:01 with lcSSc and dcSSc, respectively, while ACA was associated with HLA-DRB1*08:01 and HLA-DRB1*07:01, and ATA presented the strongest associations with HLA-DPB1*02:01 and HLA-DQB1*03:01.

### Single-cell RNA sequence for SSc

The recent advent of single-cell RNA sequencing (scRNA-seq) and technological progress have enabled us to identify or pinpoint transcriptional changes in disease-relevant tissues or cell types. A recently published systematic review of scRNA-seq studies for SSc comprehensively searched available studies published before June 23rd, 2023, and included 17 studies with high-quality evidence based on the Preferred Reporting Items for Systematic reviews and Meta-Analyses (PRISMA) guideline ^(27)^. The sources of cells are either peripheral blood, skin, or lung.

Among peripheral blood mononuclear cells, observations related to circulating monocytes (CD14^+^ or CD16^+^) could be particularly intriguing, especially in their relationship to tissue-resident macrophages ^(28)^. For example, CD14^+^ blood monocytes from SSc patients showed elevated expression of fibronectin 1 (*FN1*) and TGF-β signaling. At the same time, tissue-resident CD14^+^ pulmonary macrophages demonstrated activated profibrotic signatures, including elevated *FN1* expression in SSc patients with interstitial lung disease (ILD) ^(29)^. Such simultaneous analysis of peripheral blood and disease-relevant tissues can make it possible to identify circulating disease-specific cell populations or even track their developmental trajectories.

The lung is a major organ involved in SSc, and ILD and pulmonary arterial hypertension (PAH) are major causes of mortality. Not surprisingly, lung fibroblasts show upregulation of profibrotic genes ^(30)^, including unique expression of CTHRC-1 ^(31)^. Again, macrophages in the lung appear to play a major role in ILD development, with increased expression of osteopontin ^(32)^, type I interferon signaling ^(33)^, and profibrotic SPP1 ^(34)^, in addition to *FN1, as* described above ^(29)^. The SSc-specific contribution of SPP1 macrophages is further supported by a recent scRNA-seq study, where lung tissue samples from SSc-ILD, IPF, and HC samples were analyzed ^(35)^. Furthermore, plasmacytoid dendritic cells, CD8+ cytotoxic T cells, and natural killer cells all exhibited upregulated type I interferon (IFN) signaling in the lung, highlighting the importance of type I IFN signaling in the development of SSc-ILD ^(33), (34)^. Intriguingly, aberrant lung-specific basaloid cells and basal lung epithelial cells from SSc-ILD samples were enriched for markers of cellular senescence ^(36)^, implying a tissue-specific, non-lymphocyte pathological basis of SSc-ILD. As for SSc-PAH, only one scRNA-seq study of lung tissue from lcSSc subjects is currently available. PAH was confirmed by right heart catheterization, and those without clinical evidence of PAH were classified as controls. The study found that serum chemerin levels were significantly increased and correlated with pulmonary vascular resistance in SSc-PAH cases. Chemerin was expressed in fibroblasts, pulmonary artery smooth muscle cells (PA-SMCs)/pericytes, and mesothelial cells in SSc-PAH lungs, and chemerin receptor expression was increased in PA-SMCs of SSc-PAH ^(37)^.

Skin is a major lesion of SSc and an easily accessible location for sample collection compared to the lung or other internal organs, Hence, it has been well studied, providing valuable information on the underlying pathology of SSc. Reported skin-specific changes include the epithelial-to-mesenchymal transition of endothelial cells ^(38)^, myofibroblast differentiation of SFRP2^hi^ SSc fibroblasts ^(39)^, IFN signatures in fibroblasts ^(40)^, and plasmatoid dendritic cell (pDC) migration into the skin, which was observed almost exclusively in dcSSc samples ^(41)^. Macrophages in SSc skin highly expressed Fcγ receptor IIIA, and proliferating macrophages were detected almost exclusively in dcSSc skin ^(41)^, indicating the potential contribution of this macrophage subset to the severe form of skin lesions. Of note, the recent East Asian GWAS identified a plausible causal variant in the genomic region of the *FCGR-FCRL* gene clusters ^(21)^.

A recent skin scRNA-seq study using forearm skin biopsy samples from 12 European SSc patients and three healthy controls compared different stages of the disease (>5 or <5 years disease duration) in those with ATA and anti-RNA polymerase III antibody (ARA) subsets, referencing the healthy control samples ^(42)^. The study observed clear separation of all the cell types examined, including lymphocytes, fibroblasts, and smooth muscle cells, by disease status, stage, and antibody profile. Intriguingly, while there were many overlaps in the biological pathways involved between early-stage and late-stage disease regardless of autoantibody profiles, some pathways showed increased expression in late-stage SSc for one antibody subtype but were overexpressed in early-stage SSc for the other. Focusing on key biological pathways across all cells, genes associated with vascular endothelial growth factor pathways and extracellular matrix genes including collagen and tenascin, as well as fibrotic pathways such as *THBS* and *THY1,* were more highly expressed in the early ARA subgroup. In contrast, genes associated with MHC-I, TGFβ, NOTCH signaling, and *CD40*, *LIGHT*, and *TRAIL* were more highly expressed in the early ATA subgroup. Furthermore, using hierarchical clustering of the gene sets, cells that responded to gene sets corresponding to specific pathways were grouped with other cells responding to the same gene sets. As a notable example, fibroblasts showed the greatest response to TGFβ pathway genes in ATA patients, while such effects were mainly observed in endothelial cells within the ARA patients.

The first study, deposited on a preprint server at the time of submission of this article, investigated distinct changes in SSc esophageal epithelial cells ^(43)^. Proximal and distal esophageal biopsy samples were obtained from individuals with SSc, those with gastroesophageal reflux disease, and healthy controls. The study identified differentially expressed genes (DEGs) with significant upregulation of extracellular matrix and keratinization genes in SSc samples. Those DEGs were primarily limited to the superficial compartment (3,572 genes vs. 232 in all other compartments, based on pseudo-bulk analysis). While cellular and molecular changes in SSc were highly correlated with those seen in gastroesophageal reflux disease, the magnitudes were more pronounced in the proximal esophagus, suggesting that esophageal dysmotility leads to greater proximal acid exposure. Furthermore, cell-type localization and SSc-specific changes were confirmed by spatial molecular imaging.

While these studies successfully delineated DEGs in SSc at tissue-specific single-cell levels, such as in monocytes/macrophages or fibroblasts, many questions remained unresolved. For example, while significant heritability enrichment of active histone mark-related SNPs in B cells strongly indicates a major contribution of these cells to disease development ^(21)^, supporting evidence of scRNA-seq data was surprisingly scarce and needs to be investigated in future studies.

## Future Direction: Integrative Analysis for Better Understanding of the Disease

Large-scale GWASs, together with state-of-the-art downstream analyses, have identified multiple plausible causal signals associated with the susceptibility of not only global SSc but its clinical subtypes, mainly in individuals of European descent. While our study contributed to the advancement of GWAS for the East Asian SSc, the sample size was still much smaller than that of the European cohorts and needs to be updated with a larger sample size in the future. While rare variants have higher effect sizes than common variants, their low allele frequencies have hindered the identification of associations with disease. The recent cost reduction in the cost of whole-genome sequencing may help overcome such issues and contribute to the identification of rare variant associations. We also demonstrated the polygenic nature of SSc and the potential utility of PRS in predicting disease development, with improved performance by prioritizing more disease-relevant SNPs for PRS construction. A well-powered cross-ancestry GWAS meta-analysis and sophisticated prioritization of SNP selection will enable the construction of PRS models more suitable for stratifying subjects to predict not only disease development but also disease outcomes.

The scRNA-seq studies have contributed to a better understanding of transcriptional changes in specific cells of the tissues/organs involved, as well as potential functional consequences in a given cell. However, in most studies, the resolution of cell types and sequencing depth is still insufficient to capture rare cell populations and rare transcripts, respectively. On the other hand, more studies are beginning to investigate how genetic risks impact transcriptional changes and functional changes at the individual cell level. For example, the Single-cell Disease-Relevance Score is a method for associating individual cells in scRNA-seq data with GWASs ^(44)^. SNP2Cell provides regulatory networks linked to traits from GWAS on a per-cell type basis ^(45)^. Considering that more than 90% of GWAS risk variants are identified in regulatory elements rather than in gene bodies or coding regions, capturing the 5’-end of the transcript together with sufficient sequencing depth, enables the identification of not only promoters but also enhancers, the latter of which presents a cell type or tissue-specific pattern of expression. Cellular Indexing of Transcriptomes and Epitopes by Sequencing is a scRNA-seq method that integrates surface protein information ^(46)^. The method enables the measurement of both gene and protein expression levels, which adds another layer of information and thus enables finer cell clustering. Cap-analysis gene expression CAGE-seq allows high-throughput gene expression profiling with simultaneous identification of transcriptional start sites by specifically capturing the 5′ cap structure of newly synthesized RNAs ^(47)^. It later turns out that CAGE can also capture enhancers, which are characterized by bidirectional transcription of enhancer RNAs. Nowadays, combining CAGE-seq with other RNA capture technologies enables the efficient identification of rare cell populations based on cell type-specific enhancer expressions ^(48)^.

Integrative analysis using a multi-omics approach will dissect SSc pathology at cellular and molecular levels and lead to the development of sophisticated diagnostic tools and improved clinical management strategies based on phenotype or risk stratifications.

## Article Information

### Conflicts of Interest

None

This article is based on the study, which received the Medical Research Encouragement Prize of The Japan Medical Association in 2024.
